# Selective and Inverse U-Shaped Curve Alteration of the Retinal Nerve in Amyotrophic Lateral Sclerosis: A Potential Mirror of the Disease

**DOI:** 10.3389/fnagi.2021.783431

**Published:** 2022-01-06

**Authors:** Yixuan Zhang, Xiangyi Liu, Jiayu Fu, Yuanjin Zhang, Xue Yang, Shuo Zhang, Dongsheng Fan

**Affiliations:** ^1^Department of Neurology, Peking University Third Hospital, Beijing, China; ^2^Beijing Municipal Key Laboratory of Biomarker and Translational Research in Neurodegenerative Diseases, Beijing, China; ^3^Department of Ophthalmology, Peking University Third Hospital, Beijing, China

**Keywords:** retinal nerve fiber layer, retinal ganglion cell, amyotrophic lateral sclerosis, U-shaped curve alteration, disease progression rate

## Abstract

**Introduction:** Alterations in the visual pathway involving the retina have been reported in amyotrophic lateral sclerosis (ALS) but they lack consistency and subgroup analysis. We aimed to assess the retinal nerve fiber layer (RNFL) and retinal ganglion cells (RGCs) alterations in different stages of ALS patients and their association with ALS progression parameters.

**Methods:** The study population consisted of 70 clinically diagnosed ALS patients and 55 age, sex matched controls. All of them underwent ophthalmic assessments and optical coherence tomography imaging. Four quadrants of the peripapillary RNFL and ganglion cell/inner plexiform complex (GCIP) were observed and automatically measured. Early-stage distal motor neuron axon dysfunction in ALS was detected by compound muscle action potential (CMAP) of the distal limbs within 12 months. The ALS disease parameters included the ALSFRS-R score and the disease progression rate (ΔFS).

**Results:** Generally compared with controls, the nasal (*p* = 0.016) quadrant of the RNFL was thicker in ALS patients. When controlling for age and ΔFS, the RNFL(*r* = 0.37, *p* = 0.034) and GCIP(*r* = 0.40, *p* = 0.021) were significantly thickened as disease progressed within 12 months, while the RNFL declined with time after one year (*r* = −0.41, *p* = 0.037). ALS patients was subclassified into thickened RNFL (T-RNFL, >95th percentile of normal), impaired RNFL (I-RNFL, <5th percentile of normal) and normal RNFL. There were significant differences in the GCIP among the three groups (*p* < 0.001). In the T-RNFL group (*n* = 18), the RNFL was negatively correlated with the abductor pollicis brevis-CMAP amplitude within 12 months (*r* = −0.56, *p* = 0.01). Patients within 12 months in this group progressed faster than others (*p* = 0.039). In the normal RNFL group (*n* = 22), 13 patients were diagnosed beyond 12 months, whose ΔFS was remarkably lower (*p* = 0.007). In I-RNFL group (*n* = 30), the early stage patients (<12 months) had significant higher ΔFS (*p* = 0.006). One patient was with SOD1 pathogenic variant (p.A5V).

**Conclusion:** Alterations of retinal nerve were not consistent in ALS patients with diverse phenotypes and progression rates. Generally speaking, the RNFL thickened during the first year and then gradually declined, which is related to but preceding the thickness change of the RGCs. Patients with a significant RNFL thinning in the early stage may have a faster progression rate. The inverse U-shaped curve transformation might be in accordance with early-stage motor neuron axonopathy.

## Introduction

Amyotrophic lateral sclerosis (ALS) is a highly heterogeneous degenerative disease characterized by the deterioration of voluntary muscle function due to motor neuronal damage. However, there are some recent studies supporting the frequent involvement of other non-motor systems, including the visual pathways (Munte et al., 1998). The optic nerve and cells are unique parts of the central nervous system giving origin to 1.2 million axons and retinal ganglion cells (RGCs) and they are particularly vulnerable to neurodegeneration (Carelli et al., 2017). Moreover, optic neuropathies and ALS share important common pathogenic mechanisms, including increased levels of oxidative stress, mitochondrial damage and axonal transport disorders (Maresca et al., 2013). And a histopathological study demonstrates widespread astrogliosis in the subcortical white matter of occipital cortex in ALS (Kushner et al., 1991).

Since visual complaints are not common in ALS patients, relatively little is known about possible retinal alterations upon clinical examination. Optical coherence tomography (OCT) is a non-invasive imaging technique that is accurate, objective, and reproducible. It could provide high-resolution images of the retina and precisely and objectively quantify discrete neuronal layers, including the peripapillary retinal nerve fiber layer (RNFL, representing the unmyelinated axons of retinal ganglion cells), retinal ganglion cell bodies and the inner plexiform layer (GCIP, representing the health of the ganglion cell layer). This technique has been applied to detect neuroaxonal retinal abnormalities in neurodegenerative disease like Parkinson's Disease (Huang et al., 2020), progressive supranuclear palsy (Schneider et al., 2014), multiple system atrophy (Schneider et al., 2014) and Alzheimer's disease (Kirbas et al., 2013).

However, the reported changes in the retinal layers in ALS are not consistent. The first study performed in 76 ALS patients showed no significant difference from the controls for RNFL (Roth et al., 2013), while the majority of studies assessing RNFL found significant thinning within the ALS group (Ringelstein et al., 2014; Hubers et al., 2016; Mukherjee et al., 2017; Rohani et al., 2018; Rojas et al., 2019), with the superonasal quadrants being the most prominent (Mukherjee et al., 2017; Rohani et al., 2018; Rojas et al., 2019). However the most recent study conducted by our institution showed that the nasal quadrant of ALS RNFL even had a statistically thicker measurement (Liu et al., 2018). These studies included patients in different stages but did not stratify them according to their clinical phenotypes. We hypothesized that an alteration of a specific single retinal layer varied in ALS patients owing to disease heterogeneity.

In this study, we aimed to assess selective retinal nerve alterations in ALS patients in different stages and their associations with ALS progression parameters.

## Materials and Methods

### Participants

This study was a retrospective, cross-sectional study. Seventy patients who met the El Escorial criteria (Brooks et al., 2000) for definite, probable and possible ALS and 55 age, sex matched controls were recruited at the Department of Neurology at Peking University Third Hospital. The exclusion criteria including: (1) combining with any other neurodegenerative disorders or autoimmune inflammatory diseases of the central nervous system; (2) having any eye diseases that may involve retina (e.g., history of retinopathy, glaucoma, or high myopia); (3) combining with any other systemic diseases that affect optic nerve (such as diabetes). All patients were collected for the following variables: demographic details, symptom duration, phenotype and site of onset.

### Optical Coherence Tomography

Subjects underwent regular intraocular pressure (IOP) measurements (<20 mmHg) and were screened to exclude central neurologic disorders or ocular diseases other than glaucoma that may affect the OCT results.

SD-OCT was performed using a Spectralis setup with TruTrackimage alignment eye tracking (Heidelberg Engineering, Heidelberg, Germany). The peripapillary RNFL and macula were measured in all cases. The optic nerve head was automatically scanned to determine the RNFL thickness map. The temporal, superior, nasal and inferior quadrants of the RNFL as well as the average thickness measurements were analyzed. Semi-automated retinal segmentation software (Eye Explorer package Heidelberg Engineering; version: 1.10.4.0) was used to quantify and evaluate the macular sublayers, including ganglion cell bodies and the inner plexiform layer, which are combined into GCIP (Hogan and Weddell, 1971).

The patients were subdivided into three groups according to their OCT scans. Group 1 consisted of patients who demonstrated a predominantly thickened RNFL (T-RNFL), which were defined as having an RNFL combination of >95th percentile of normal. Group 2 included patients with a normal RNFL, whose OCT combination was within the normal range (5th−95th percentile). Group 3 was comprised of patients with a significantly impaired retinal layer (I-RNFL), whose OCT scans were <5th percentile of normal.

### Clinical Examination

The disease course of patients were staged by King's College staging system (KCSS) (Roche et al., 2012). The revised ALS Functional Rating Scale (ALSFRS-R) (Cedarbaum et al., 1999) was used to assess the functional status of the patients, which is a self-report questionnaire consisting of 12 questions evaluating the bulbar, motor and respiratory functions of ALS patients. Each question is scored between 0 and 4 (four being totally intact and 0 meaning total loss of function) (Bakker et al., 2017), with higher scores meaning more retained function. The disease progression rate (ΔFS) was calculated using the following formula (Kimura et al., 2006): 48-total ALSFRS-R at assessment/symptom duration (months). The severity of the upper motor neuron (UMN) damage was graded using a modified Ravits Scale (Ravits et al., 2007).

### Compound Muscle Action Potential (CMAP) Examination

The amplitude of the compound muscle action potential (CMAP) is a widely used electrophysiological index to detect motor axonal damage (Liu et al., 2009). In our study, degeneration of the terminal motor axons was evaluated by the CAMP recorded from the abductor pollicis brevis (APB) and extensor digitorum brevis (EDB) stimulation. Nerve conduction studies were carried out with Keypoint four-channel electromyography evoked potentiometer (Medtronic, USA). Motor nerve conduction of the median nerves and peroneal nerves was examined by routine methods. CMAP amplitudes of the most severely affected side of the patients were recorded, which were also scored according to the published stratification criteria (Yu et al., 2021): 0 point (normal): ≥the lower limit of normal (LLN); 1 point (mild decrease): < LLN but ≥50% of LLN; 2 points (moderate decrease): <50% but ≥30% of LLN, and 3 points (severe decrease): <30% of LLN (Supplementary Table 1).

### Ethics

All participating patients and controls provided written informed consent for the OCT protocol according to institutional guidelines. This study was approved by the Ethical Committee of Peking University Third Hospital (approval No. M2019388) and was conducted in accordance with the Declaration of Helsinki.

### Statistical Analysis

Statistical analysis was carried out using SPSS (Statistical Package for Social Sciences) Windows version 23.0. The normal distribution of the variables was checked using the Kolmogorov–Smirnov test. Data are presented as the mean ± SD for normally distributed data. Non-normally distributed data are given in medians and quartiles. Data conforming to a normal distribution were tested using the independent sample *t*-test for two group analysis, and tested by univariate ANOVA for three-group analysis. Data that did not conform to a normal distribution were compared by the Mann-Whitney U test for two group analysis and the Kruskal-Wallis test for multiple group analysis. We used partial bivariate correlation analysis corrected for age and ΔFS to analyze the associations between the retinal parameters in the ALS patients and the disease duration as well as the CMAP amplitudes. A *p* value < 0.05 was considered significant.

## Results

### Patient Demographics

OCT scans from 70 ALS patients and 55 healthy controls were included in the analyses. ALS patients and healthy controls did not differ in age or sex. The disease duration ranged from 1 month to 72 months, while the median time was 13.8 months. Among our patients, 17% were bulbar onset, and 83% were spinal onset. Diagnostic grades included 20 patients (28.6%) as definite, 27 patients (38.6%) as probable, 23 patents (32.9%) as possible ALS. Regarding disease progression graded by KCSS, 44.3% of patients were in stage 1, 28.6% were in stage 2, 18.6% were in stages 3 and 8.6% were in stage 4. One patient was with SOD1 pathogenic variant (p.A5V) (Table 1).

**Table 1:** The demographics and general clinical characteristics of the ALS patients and healthy controls.

**Characteristics**	**ALS (*n* = 70)**	**Healthy Controls (*n* = 55)**	***p***
Age (y)	53.80 ± 10.95	52.4 ± 12.7	0.539
Male sex	47 (67.1%)	29 (52.7%)	0.073
KCSS			
1	31 (44.3%)		
2	20 (28.6%)		
3	13 (18.6%)		
4	6 (8.6%)		
ALSFRS-R	39.56 ± 5.87	N/A	
Disease onset location		N/A	-
Bulbar	12 (17.1%)		-
Spinal-upper	36 (51.4%)		-
Spinal-lower	22 (31.4%)		-
Diagnostic level		N/A	
Definite	20 (28.6%)		
Probable	27 (38.6%)		
Possible	23 (32.9%)		

*KCSS, King's College staging system; ALSFRS-R, revised ALS Functional Rating Scale*.

### OCT in ALS vs. Health Controls

In those who underwent a complete ophthalmic assessments, there was no evidence of optic disc swelling or macular edema. Funduscopic examination did not reveal any clinical signs of vasculitis, vitritis (no vitreous haze or cells) or intermediate uveitis (snow balls, snow banking or exudates in the peripheral retina and pars plana).

The overall OCT analysis—disregarding laterality—in 70 ALS and 55 healthy eyes, showed a significantly thickened RNFL thickness globally (*p* = 0.048), and the RNFL in the nasal quadrant was mostly thickened (*p* = 0.016). While no significant differences were found in GCIP between patients and controls (*p* = 0.243). Within-case comparisons showed significant differences in RNFL (*p* < 0.001), GCIP (*p* < 0.001), KCSS (*p* = 0.005) and ΔFS (*p* = 0.020) among the three groups (Table 2).

**Table 2:** Comparison of the OCT results and ALS disease parameters among the groups.

	**Case/control comparison**			**Within-case comparison**			
	**ALS (*n* = 70)**	**Healthy controls** **(*n* = 55)**	***p***	**T-RNFL** **(*n* = 18)**	**Normal RNFL (*n* = 22)**	**I-RNFL** **(*n* = 30)**	***p***
RNFL (μm)							
S	123.48 ± 15.70	125.27 ± 9.98	0.441	134.69 ± 14.42	124.95 ± 8.28	114.90 ± 16.17	0.003**
I	132.97 ± 17.91	127.86 ± 16.90	0.108	149.39 ± 14.58	128.14 ± 11.22	124.17 ± 17.52	<0.001***
T	75.84 ± 12.78	73.04 ± 9.25	0.175	77.83 ± 8.51	74.81 ± 12.11	72.62 ± 14.87	0.283
N	68 (61–78)	63.36 ± 11.98	0.016*	78.50 ± 13.66	71.36 ± 9.94	61.93 ± 11.72	<0.001***
Global	100.48 ± 10.97	97.38 ± 6.00	0.048*	110.10 ± 8.80	100.59 ± 7.08	94.21 ± 10.23	<0.001***
GCIP (μm)	72.21 ± 7.92	73.38 ± 2.42	0.243	76.40 ± 5.94	74.57 ± 5.18	67.96 ± 8.68	<0.001***
Age (y)	53.80 ± 10.95	52.4 ± 12.7	0.539	52.61 ± 10.77	54.18 ± 11.59	55.79 ± 10.39	0.070
Disease duration (m)	18.92 ± 15.36	N/A		15.52 ± 10.76	17.94 ± 12.84	21.67 ± 18.93	0.387
KCSS							0.005**
1	31 (44.3%)			6 (33.3%)	12 (54.5%)	13 (43.3%)	
2	20 (28.6%)			8 (44.4%)	5 (22.7%)	6 (20%)	
3	13 (18.6%)			4 (22.2%)	3 (13.6%)	6 (20%)	
4	6 (8.6%)			0	2 (9.1%)	4 (13.3%)	
CMAP score	2.59 ± 1.83	N/A		3.06 ± 1.89	1.67 ± 1.76	2.83 ± 1.67	0.065
UMN score	5.61 ± 3.54	N/A		5.17 ± 3.45	6.23 ± 3.58	5.43 ± 3.62	0.605
ΔFS	0.5 (0.25–1)	N/A		0.71 (0.38–1.13)	0.31 (0.21–0.54)	0.54 (0.27–1.00)	0.020*

*Normally distributed data are presented as the mean ± SD, and non-normally distributed data are presented as medians (IQRs).*

*RNFL, retinal nerve fiber layer; S, superior; I, inferior; T, temporal; N, nasal; GCIP, ganglion cell/inner plexiform; KCSS, King's College staging system; CMAP, compound muscle action potential; UMN, upper motor neuron; ΔFS, 48-(ALSFRS-R at assessment)/symptom duration (months).*

*p ≤ 0.05, **p ≤ 0.01, ***p < 0.001.

### OCT in ALS vs. Disease Progression

**Figure 1:**
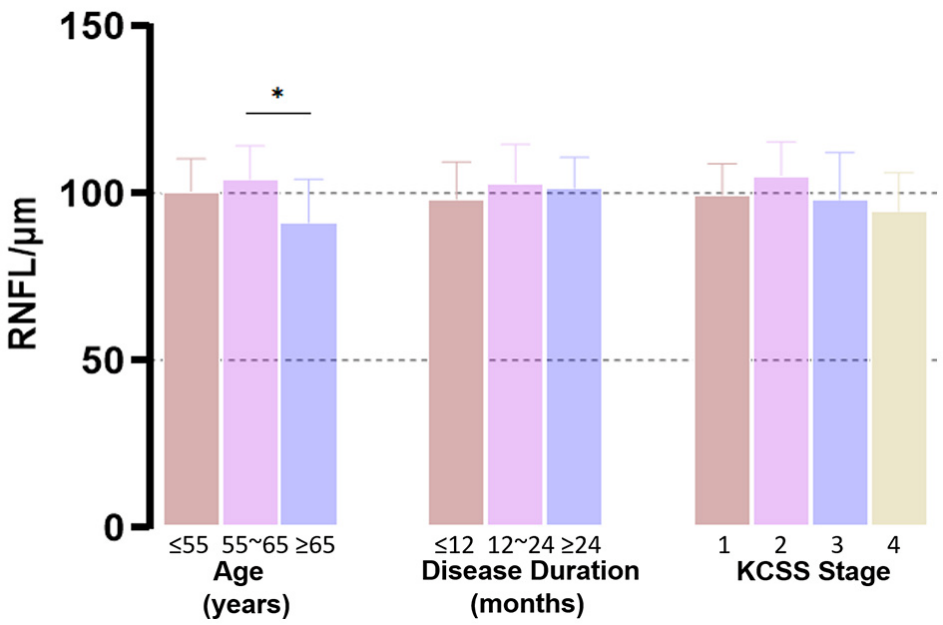
Group-wise comparison of RNFL. ALS patients were grouped according to age, disease duration and KCSS. We detected a significantly thinner RNFL in older (≥65 years) subjects (*p* = 0.020). There were no significant differences between groups when stratified by disease duration (*p* = 0.395) or KCSS (*p* = 0.234). *p ≤ 0.05.

In order to evaluate the association between RNFL and disease progression, we applied two approaches. First, we stratified ALS patients according to age, disease duration and KCSS. We detected a significantly thinner RNFL in older (≥65 years) than younger subjects (*p* = 0.020), especially on superior quadrant (*p* = 0.017). There were no significant differences between groups when stratified by disease duration (*p* = 0.395) or KCSS (*p* = 0.234) (Figure 1).

**Figure 2:**
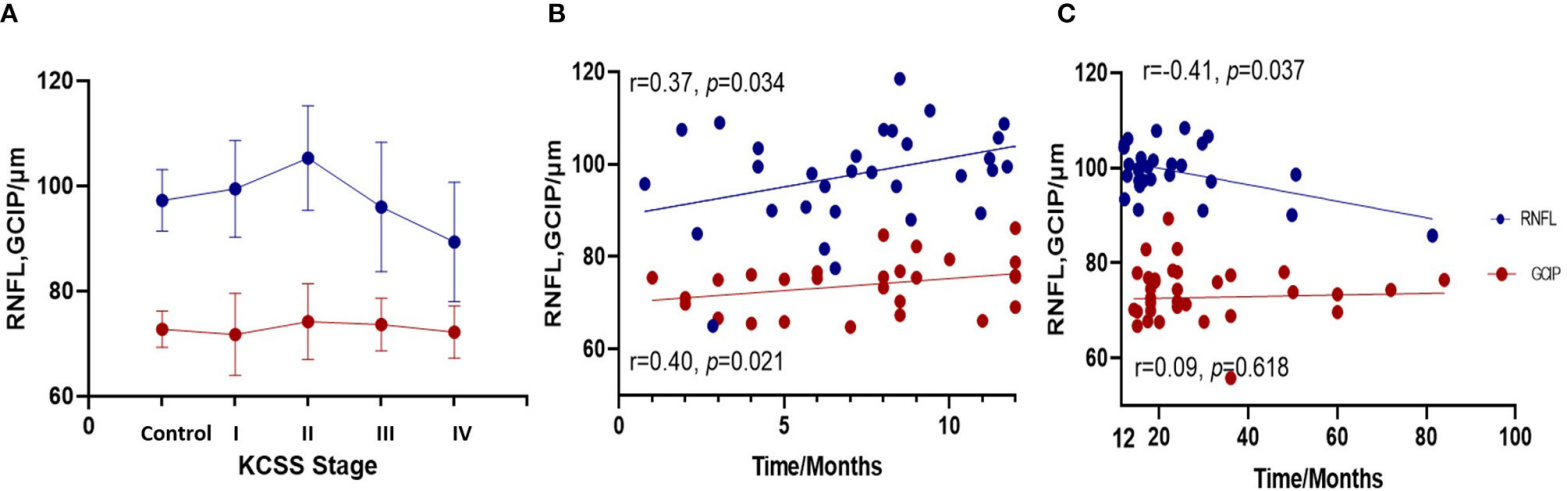
Correlation analysis between disease duration and RNFL and GCIP. **(A)** When patients were divided into four groups by disease progression based on KCSS, the RNFL remarkably increased (*r* = 0.464, *p* < 0.001) during the early stage and then significantly declined as the disease progressed (*r* = −0.324, *p* = 0.047), forming an inverse U-shaped trajectory. **(B)** In the first 12 months, the RNFL and GCIP thickened as the disease progressed (*r* = 0.37, *p* = 0.034). **(C)** After 12 months, the RNFL declined (*r* = −0.41, *p* = 0.037), but no significant correlation was found between the GCIP and disease duration.

Next, we conducted correlation analysis. When patients were divided into four groups by disease progression based on KCSS, the RNFL significantly increased (*r* = 0.464, *p* < 0.001) during the early stage and this was followed by a decline (*r* = −0.324, *p* = 0.047) as the disease progressed, forming an inverse U-shaped trajectory (Figure 2A). But this changing trend was not evident for GCIP. Partial bivariate correlation analysis (correcting for age and ΔFS) for the ALS patients revealed a significant thickening of the RNFL (*r* = 0.37, *p* = 0.034) and GCIP (*r* = 0.40, *p* = 0.021) with disease progression within 12 months, while the RNFL declined with time (*r* = −0.41, *p* = 0.037) after one year. No significant correlation was found between GCIP and disease duration 12 months later (*r* = 0.09, *p* = 0.618) (Figures 2B,C).

### T-RNFL Group

**Figure 3:**
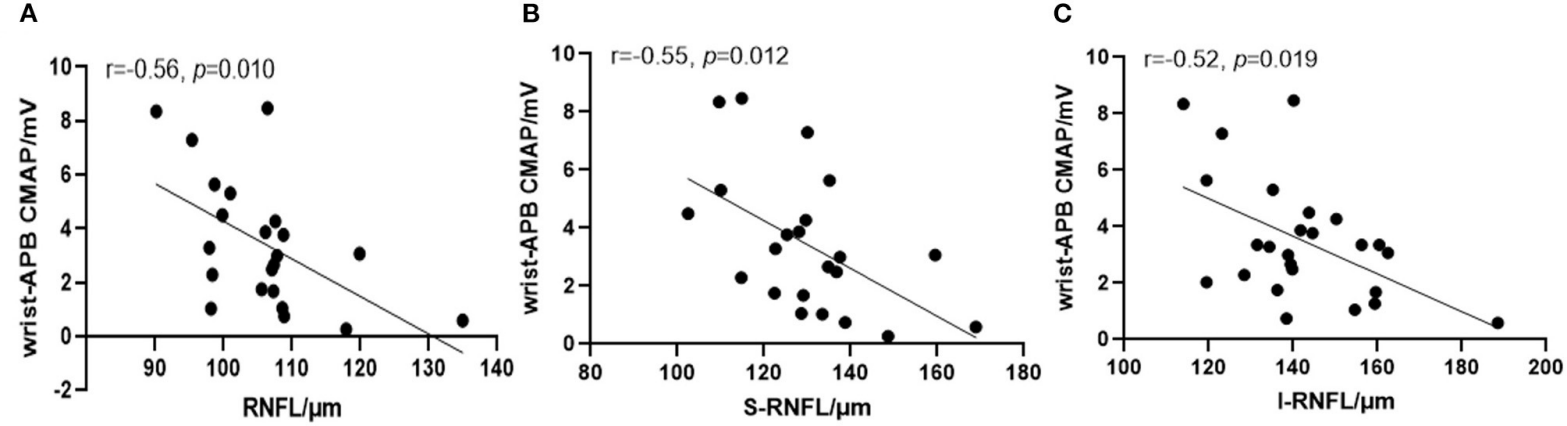
Partial correlation analysis between RNFL and the CMAP amplitude recorded from the abductor pollicis brevis after median nerve stimulation. The global **(A)**, superior **(B)** and inferior **(C)** quadrants of the RNFL thickened significantly as the abductor pollicis brevis (APB)-CMAP amplitude declined.

In the T-RNFL group, the RNFL and GCIP were significantly thickened compared with other ALS patients (Table 3). Early-stage distal motor neuron axon dysfunction in ALS was detected by the compound muscle amplitude potential (CMAP) amplitude of the distal limbs within 12 months. In the partial bivariate correlation analysis (controlling for ΔFS and the site of onset), we included normal RNFL and T-RNFL patients, and it was shown that in the first 12 months, the RNFL thickened significantly as the APB-CMAP amplitude declined (Figure 3). The patients within 12 months in this group progressed faster than others (*p* = 0.039).

**Table 3:** Comparison of RNFL, GCIP and disease parameters between the T-RNFL group patients and the other ALS patients.

	**T-RNFL (*n* = 18)**	**Other ALS patients (*n* = 52)**	***p***
RNFL (μm)	110.10 ± 8.80	96.96 ± 9.48	<0.001***
GCIP (μm)	76.40 ± 5.94	70.76 ± 8.05	0.008**
Wrist-APB CMAP (mV)	2.40 ± 2.94	4.08 ± 3.15	0.100
Ankle-EDB CMAP (mV)	2.81 ± 2.30	2.23 ± 1.73	0.401
CMAP score	3.06 ± 1.89	2.37 ± 1.78	0.191
UMN score	5.17 ± 3.45	5.77 ± 3.59	0.538
ΔFS			0.037*
Within 12 months (*n* = 10)	0.94 (0.43–1.96)	0.41 (0.23–0.90)	0.039*
After 12 months (*n* = 8)	0.58 (0.36–0.91)	0.41 (0.23–0.90)	0.276

*RNFL, retinal nerve fiber layer; GCIP, ganglion cell/inner plexiform; APB, abductor pollicis brevis; EDB, extensor digitorum brevis; CMAP, compound muscle action potential; UMN, upper motor neuron; ΔFS, 48-(ALSFRS-R assessment)/symptom duration (months).*

*p ≤ 0.05, **p ≤ 0.01, ***p < 0.001.

### Normal RNFL Group

Of those with a normal RNFL in all four quadrants, CMAP scores (*p* = 0.021) and ΔFS (*p* = 0.011) were significantly lower than in the other ALS patients with abnormal OCT (Table 4). Notably, 13 patients (59.1%) in this group were diagnosed more than 12 months prior. They belonged to variants with a benign natural course, comprising three with flail arm syndrome, two with flail leg syndrome, three with isolated bulbar palsy and two with primary lateral sclerosis, whose ΔFS was remarkably lower than that of patients with abnormal OCT (*p* = 0.007).

**Table 4:** Comparison of RNFL, GCIP and disease parameters between patients with normal RNFL and the other ALS patients.

	**Normal RNFL (*n* = 22)**	**Other ALS patients (*n* = 48)**	***p***
RNFL (μm)	100.59 ± 7.08	100.30 ± 12.38	0.902
GCIP (μm)	74.57 ± 5.18	71.13 ± 8.74	0.092
Wrist-APB CMAP (mV)	4.50 ± 3.56	3.28 ± 2.94	0.216
Ankle-EDB CMAP (mV)	2.16 ± 1.07	2.49 ± 2.14	0.480
CMAP score	1.67 ± 1.76	2.93 ± 1.75	0.021*
UMN score	6.23 ± 3.58	5.33 ± 3.52	0.614
ΔFS			0.011*
Within 12 months (*n* = 9)	0.38 (0.21–1.15)	0.58 (0.31–1.00)	0.288
After 12 months (*n* = 13)	0.28 (0.19–0.45)	0.58 (0.31–1.00)	0.007**

*RNFL, retinal nerve fiber layer; GCIP, ganglion cell/inner plexiform; APB, abductor pollicis brevis; EDB, extensor digitorum brevis; CMAP, compound muscle action potential; UMN, upper motor neuron; ΔFS, 48-(ALSFRS-R assessment)/symptom duration (months).*

*p ≤ 0.05, **p ≤ 0.01.

### I-RNFL Group

The average age of the patients in the I-RNFL group was higher albeit not significant (Table 5). Among them, 36.7% were definite ALS. In the partial correlation analysis correcting for ΔFS, the UMN score fell significantly with the decrease of RNFL (*r* = 0.44, *p* = 0.016).

**Table 5:** Comparison of RNFL, GCIP and disease parameters between the I-RNFL patients and the other ALS patients.

	**I-RNFL (*n* = 30)**	**Other ALS patients (*n* = 40)**	***p***
Age	55.79 ± 10.39	52.44 ± 11.24	0.215
RNFL	94.21 ± 10.23	104.23 ± 8.63	<0.001***
GCIP	67.96 ± 8.68	75.42 ± 4.79	<0.001***
Wrist-APB CMAP	4.11 ± 2.89	3.31 ± 3.36	0.387
Ankle-EDB CMAP	2.62 ± 2.13	2.24 ± 1.75	0.496
CMAP score	2.83 ± 1.67	2.83 ± 1.82	0.209
UMN score	4.82 ± 3.94	5.81 ± 3.44	0.324
ΔFS	0.54 (0.27–1.00)		0.826
Within 12 months (*n* = 15)	1.00 (0.55–1.50)	0.50 (0.26–1.00)	0.006**
After 12 months (*n* = 15)	0.28 (0.18–0.53)	0.50 (0.26–1.00)	0.057

*RNFL, retinal nerve fiber layer; GCIP, ganglion cell/inner plexiform; APB, abductor pollicis brevis; EDB, extensor digitorum brevis; CMAP, compound muscle action potential; UMN, upper motor neuron; ΔFS, 48-(ALSFRS-R assessment)/symptom duration (months).*

**p ≤ 0.01, ***p < 0.001.

We did not find any significant distinction in CMAP, UMN score or ΔFS between I-RNFL and the others, because this group still contained patients in diverse stages (50% were diagnosed within 12 months, while the others were diagnosed beyond 12 months). However, for early stage patients (<12 months) with I-RNFL (*n* = 15), their ΔFS was much higher than that of the other patients (*p*=0.006). Among them, one patient was with SOD1 pathogenic variant (p.A5V).

## Discussion

The retina is a highly structured tissue sharing embryonic commonality with central nervous system. It can be divided into 10 layers histologically. Photoreceptors transmit light stimulus to RGCs (Cervero et al., 2021), the neuronal cell type that projects its axon to the brain, forming the optic nerve (Carelli et al., 2004; Yu-Wai-Man et al., 2011). RGCs are the usual target in neuropathies implying an impaired mitochondrial function (Maresca et al., 2013), and this vulnerability is attributed to the unique axonal structure, characterized by a long intraretinal segment that remains unmyelinated (Carelli et al., 2004; Yu-Wai-Man et al., 2011). Since mitochondrial dysfunction is a major event in the pathology of ALS (Cozzolino and Carri, 2012), the degree of retinal thinning in ALS patients has been recently studied, with promising but discordant results. This retrospective, cross-sectional study enrolling 70 ALS patients demonstrated for the first time that (1) the RNFL is selectively targeted in specific patients; (2) when controlling for patient age and ΔFS, the RNFL formed an inverse U-shaped curve transformation, which is related to but not fully concordant with the thickness change of the RGCs; (3) The thickening of RNFL may be related to peripheral nerve motor axonal damage.

In group-wise comparisons, age related decline of RNFL thickness that has been previously reported (Parikh et al., 2007; Leung, 2012; Hondur et al., 2018) was also observed in our study. Thus age was included as a controlling variable in the relevant analysis. Due to the disequilibrium in sample size and variance between each group, we did not find statistical differences in RNFL when patients were grouped by disease duration or KCSS.

**Figure 4:**
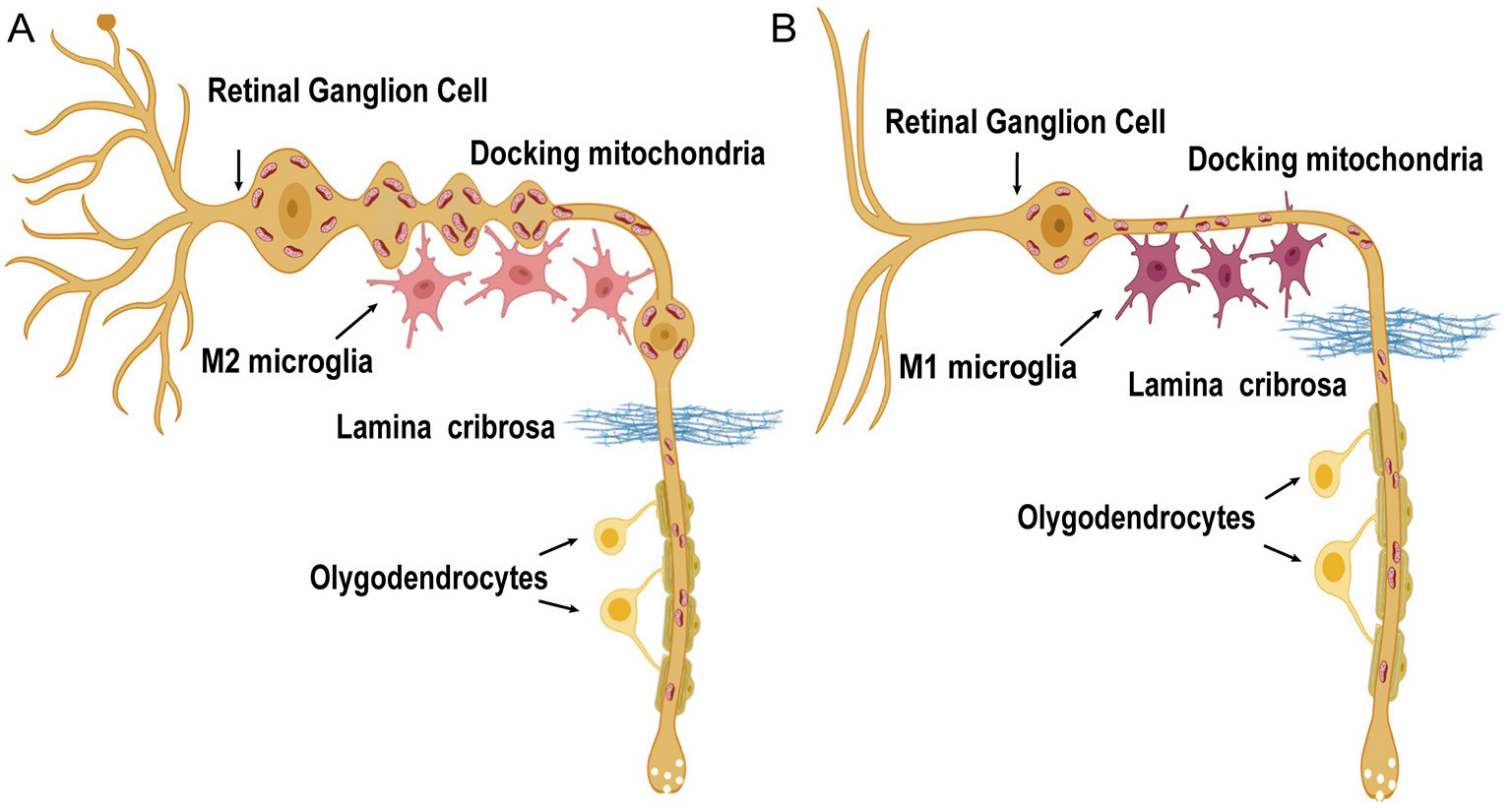
Diagrammatic view of the pathogenic mechanisms in RNFL and GCIP degeneration in ALS patients. RGCs are characterized by unique long axonal segments running unmyelinated in the RNFL before these fibers cross the lamina cribrosa. Axonal mitochondria move bidirectionally along microtubule tracks and distribute asymmetrically in the RNFL. **(A)** In the early phase, preceding the loss of macular RGCs and RNFL axons, axonal swelling is observed. The M2 microglia predominate. **(B)** As the disease progresses, proinflammatory M1-type microglia are more common. Retinal nerve fibers degenerate together with the dendrites of RGCs [the schematic figure of the RGC is modified from Carelli et al. (2004)].

In the relevant analysis, we found that the nasal and global quadrants of the RNFL in ALS patients were significantly thickened, which is consistent with previous studies (Liu et al., 2018; Rojas et al., 2019). According to the longitudinal study conducted by Rojas et al. (2019), there is a remarkable thickness evolution from increased to thinning in both the macula and the RNFL. Notably, we demonstrated that the turning point of the alteration curve might be approximately 12 months, which has been regarded as a relatively early stage of the disease (Chio et al., 2013; Marin et al., 2016; Gille et al., 2019). In both animal models and ALS patients, neuroinflammation status was more prominent during the initial 12 months (Chen et al., 2019), and neuroprotection was indicated to be the major modulation (Chiu et al., 2008; Henkel et al., 2013; Ehrhart et al., 2015). As an extension of the brain, the same process occurs in the retina (Ramirez et al., 2017), and microglial cells intermingling between RGCs play a key role (Glass et al., 2010). Activated microglia displayed retraction of processes and enlargement of the soma, resulting in the increased thickness of RGCs. Moreover, mitochondrial dysfunction in ALS could be responsible for calcium homeostasis disruption and axonal transport impairment (De Vos and Hafezparast, 2017), which is characterized by axonal swelling due to stalling of transport (Carelli et al., 2004; Yu-Wai-Man et al., 2011). These observations suggested that in the early stage of ALS, neuroinflammatory activation of microglia and an increase in axonal caliber could produce the increased thickness observed by OCT. As the disease progresses, microglial activation switches from a neuroprotective M2 to a toxic M1 phenotype (Liao et al., 2012; Chiu et al., 2013). In this state, microglia release neurotoxic inflammatory factors that lead to retinal neuronal death (Rojas et al., 2021) (Figure 4). Additionally, Rohani et al. (2018) suggested that the thinning of the RNFL could be related to Wallerian degeneration secondary to the death of cortical neurons.

The canonical view is that RGC degeneration may affect the soma after axonal involvement in a retrograde march (Carelli et al., 2004). In our study, GCIP was in accordance with the RNFL thickness in each group and it thickened significantly along with the RNFL in the initial 12 months. Nevertheless, we did not detect a decline in GCIP thickness similar to that of the RNFL one year later, indicating that the earlier pathological events may involve axons of nerve fibers preceding RGCs loss, which suggests a “dying-back” degeneration pattern (Dadon-Nachum et al., 2011) in the retina of ALS.

To detect the association between early-stage axonal degeneration of retinal nerve fibers and peripheral motor neurons, we assessed the CMAP amplitude of the distal motor nerve and calculated the CMAP decline score. In the T-RNFL group, the CMAP score was higher although not significant. Notably, when controlling for ΔFS and the site of onset, the thickening of the RNFL was significantly correlated with the decline in APB-CMAP within 12 months. From our previous research, the discrepancy in the CMAP was obvious at 12 months (Yu et al., 2021), and the decline in the CMAP at the distal median nerve within 12 months can reflect peripheral nerve axonal injury and indicate disease prognosis outside the onset site (Eisen and Kuwabara, 2012; Imai et al., 2020; Yu et al., 2021). In our study, we also found that patients in the T-RNFL group within 12 months progressed faster than others. Dibaj et al. (2011) found that during the course of microglia-mediated neuroinflammation in the central nervous system, degenerated lower motor axons were also surrounded by phagocytic phenotype macrophages. Therefore, early stage RNFL thickening might be related to the axonal inflammatory response and axonopathy. In the I-RNFL group, RGCs and peripheral motor neurons were evidently lost, and deep tendon reflexes were hard to induce in the clinically weak and wasted muscles. In this scenario, we found that the UMN score declined significantly with the thinning of the RNFL.

Our study showed that there was significant heterogeneity in RNFL changes among different ALS patients. This type of selective alteration may be related to the patients' genotype, metabolism *in vivo*, environment *in vitro* and the expression level and spread rate of pathogenetic proteins. RNFL thinning in the early stage may indicate a significantly faster deterioration (Table 5); however, patients whose RNFL was still unaffected after one year may have a relatively slower rate of progression (Table 4). The selective alteration of the RNFL, especially in the early stage, could be a new non-invasive biomarker to predict disease progression.

With the burgeoning OCT analysis in ALS patients, our findings about the retinal nerve fibers may represent a unique susceptibility of the RNFL to ALS pathology. However, there are several limitations to this study. Although we controlled for age and ΔFS to minimize the heterogeneity of the disease in correlation analysis, this is still a retrospective, cross-sectional study. A follow-up evaluation will be more convincing to prove the alteration trend. Second, although previous studies have detected alterations of the early components of the visual evoked potentials (VEPs) (Munte et al., 1998), we did not record VEPs in our cohorts, since this examination is time-consuming and requires cooperation from the patient. Third, the relationship between RNFL thickness and magnetic resonance imaging (MRI) measurements of optic nerve and brain structure volumes was not detected in our study. Since previous studies have reported the association between RNFL thickness and the volumes of brain regions vulnerable to aging or neurodegeneration (Shi et al., 2020), more imaging parameters are expected to be included in future studies. Further, we did not take macular layers such as the inner nuclear layer, outer plexiform layer or outer nuclear layer into account. Kim et al. (2019) found progressive outer retinal thinning in frontotemporal degeneration (FTD) patients. Given that up to 13% of ALS cases are associated with coincident FTD (Phukan et al., 2012), it is noteworthy to analyze the cognitive impairment and outer retinal thickness in this cohort in the future.

Visual symptoms are not a prominent complaint in most patients with ALS and can be easily concealed by motor system involvement. Our data support previously reported clinical and neurophysiological studies on visual disturbances in ALS (Rojas et al., 2019) and indicate a neurodegenerative process involving the retina. According to the four pTDP-43 pathology propagation stages (Brettschneider et al., 2013), occipital cortex and retinal axons rarely showed pTDP-43 inclusions in early stage, thus RNFL thinning in the early stage may indicate a faster propagation and deterioration. This inverse U-shaped curve transformation might be due to the initial priming and then inflammatory reaction of microglia intermingling between retinal nerve axons, and the RNFL thickening may be related to the early axonal inflammatory response. In this scenario, retinal changes emerge as a complementary window to approach the disease and can be used to assess disease progression in ALS patients.

## Data Availability Statement

The original contributions presented in the study are included in the article/Supplementary Material, further inquiries can be directed to the corresponding author.

## Ethics Statement

The studies involving human participants were reviewed and approved by the Ethical Committee of Peking University Third Hospital (approval No. M2019388). The patients/participants provided their written informed consent to participate in this study.

## Author Contributions

YiZ and DF designed the research. YiZ wrote the main manuscript text. YiZ, XL, and JF performed data analysis and interpreted the findings. YuZ and SZ contributed to the recruitment and evaluation of patients. XY contributed to the acquisition of imaging data. All of the authors read the draft, made contributions, and approved the final manuscript.

## Funding

This study was supported by the National Natural Science Foundation of China (81873784 and 82071426) and Clinical Cohort Construction Program of Peking University Third Hospital (BYSYDL2019002).

## Conflict of Interest

The authors declare that the research was conducted in the absence of any commercial or financial relationships that could be construed as a potential conflict of interest.

## Publisher's Note

All claims expressed in this article are solely those of the authors and do not necessarily represent those of their affiliated organizations, or those of the publisher, the editors and the reviewers. Any product that may be evaluated in this article, or claim that may be made by its manufacturer, is not guaranteed or endorsed by the publisher.
