# Supplementary material for: Selective and Inverse U-Shaped Curve Alteration of the Retinal Nerve in Amyotrophic Lateral Sclerosis: A Potential Mirror of the Disease
Source: Front Aging Neurosci. 2022 Jan 6;13:783431. doi: 10.3389/fnagi.2021.783431 (PMC8770270; doi:10.3389/fnagi.2021.783431)

Supplementary Material

**Supplementary Table 1.** The stratification criteria of abductor pollicis brevis-CMAP and extensor digitorum brevis-CMAP.

| **Nerve** | **0 (Normal)** | **1 (mild decrease)** | **2 point (moderate decrease)** | **3 (severe decrease)** |
| --- | --- | --- | --- | --- |
| Median nerve (mV) |  |  |  |  |
| ≤35-44 (year) | ≥5.270 | [2.635, 5.270) | [1.581, 2.635) | <1.581 |
| 45-64 (year) | ≥4.650 | [2.325, 4.650) | [1.395, 2.325) | <1.395 |
| 65-74 (year) | ≥4.480 | [2.240, 4.480) | [1.344, 2.240) | <1.344 |
| Peroneal nerve (mV) | ≥2 | [1.000, 2.000) | [0.600, 1.000) | <0.600 |

**Supplementary Figure 1.** RNFL thickness maps and APB-CMAP waveforms in ALS patients. (A) A patient in the normal-RNFL group with 20 months of disease duration, ΔFS=0.40 and a normal median nerve CMAP waveform; (B), (C) Patients in the T-RNFL within 12 months of disease duration, ΔFS >1, whose median nerve CMAP amplitude decreased at an early stage; (D) A patient in the I-RNFL with 10 months of disease duration, DPI= 1, whose median nerve CMAP was not able to be elicited.


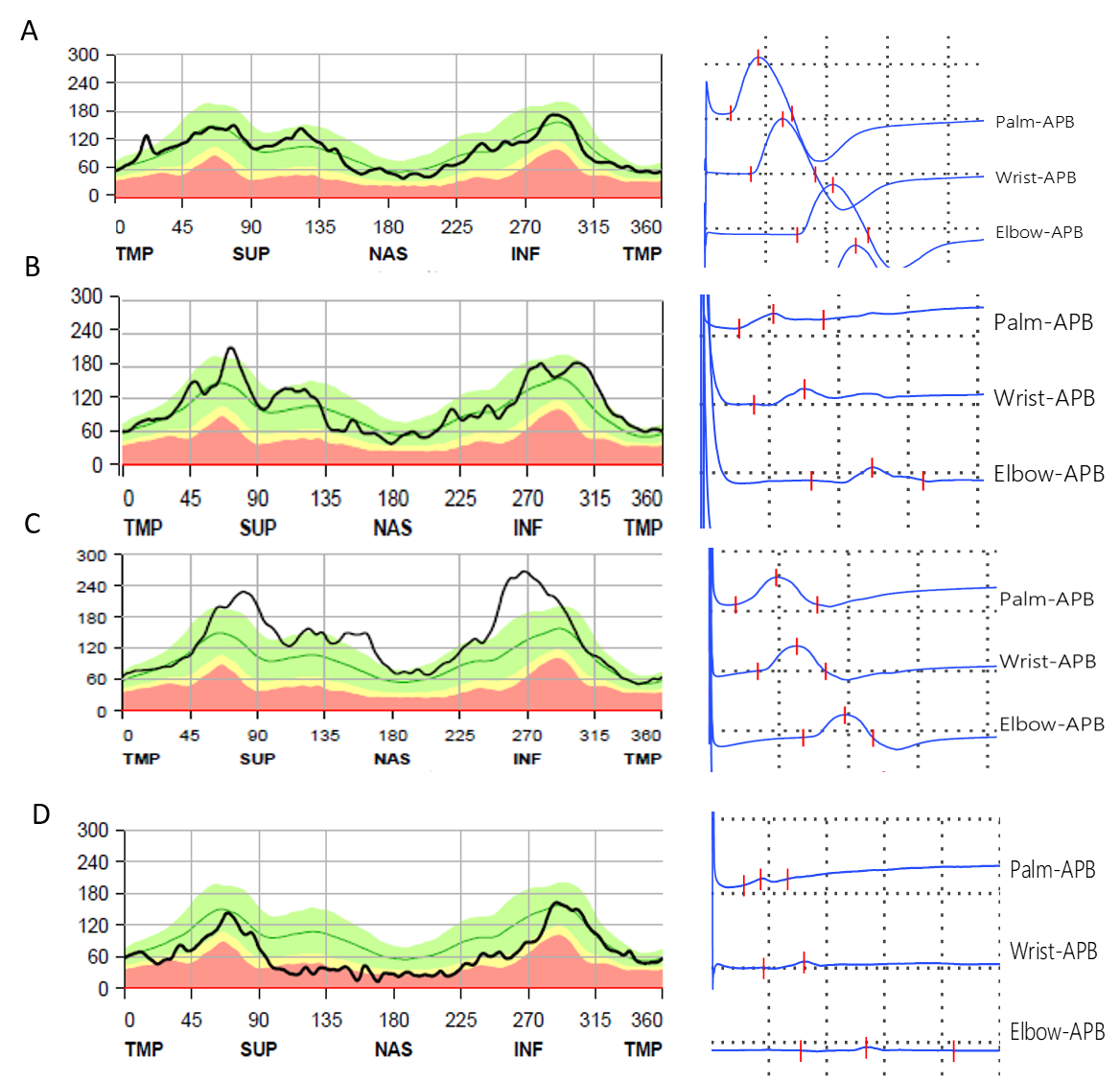

Supplement: Supplementary file 1 [file Data_Sheet_1.DOCX]
